# Specific Microbial Communities Are Selected in Minimally-Processed Fruit and Vegetables according to the Type of Product

**DOI:** 10.3390/foods11142164

**Published:** 2022-07-21

**Authors:** Giuseppina Sequino, Vincenzo Valentino, Elena Torrieri, Francesca De Filippis

**Affiliations:** 1 Department of Agricultural Sciences, University of Naples Federico II, Via Università 100, 80055 Portici, Italy; giuseppina.sequino@unina.it (G.S.); vincenzo.valentino2@unina.it (V.V.); elena.torrieri@unina.it (E.T.); 2 Task Force on Microbiome Studies, University of Naples Federico II, Corso Umberto I 40, 80138 Naples, Italy

**Keywords:** fruit microbiota, vegetable microbiota, vegetable spoilage, fresh-cut, minimally-processed vegetables

## Abstract

Fruits and vegetables (F&V) products are recommended for the daily diet due to their low caloric content, high amount of vitamins, minerals and fiber. Furthermore, these foods are a source of various phytochemical compounds, such as polyphenols, flavonoids and sterols, exerting antioxidant activity. Despite the benefits derived from eating raw F&V, the quality and safety of these products may represent a source of concern, since they can be quickly spoiled and have a very short shelf-life. Moreover, they may be a vehicle of pathogenic microorganisms. This study aims to evaluate the bacterial and fungal populations in F&V products (i.e., iceberg lettuces, arugula, spinaches, fennels, tomatoes and pears) by using culture-dependent microbiological analysis and high-throughput sequencing (HTS), in order to decipher the microbial populations that characterize minimally-processed F&V. Our results show that F&V harbor diverse and product-specific bacterial and fungal communities, with vegetables leaf morphology and type of edible fraction of fruits exerting the highest influence. In addition, we observed that several alterative (e.g., *Pseudomonas* and *Aspergillus*) and potentially pathogenic taxa (such as *Staphylococcus* and *Cladosporium*) are present, thus emphasizing the need for novel product-specific strategies to control the microbial composition of F&V and extend their shelf-life.

## 1. Introduction

Fruits and vegetables (F&V) comprise an essential part of the human diet as they are a major source of dietary nutrients and extremely important for a balanced diet. Advances in agronomic, processing, distribution and preservation techniques allowed the supply of nearly all types of high-quality fresh F&V, as well as to extend their shelf-life [1].

Despite the benefits derived from eating raw F&V, quality and safety are still an issue, since these products have an extremely short shelf-life, can be quickly spoiled and represent a potential vehicle of pathogens [2,3]. Indeed, raw vegetables are among the most frequent causes of foodborne outbreaks [4,5,6]. A first microbial contamination may generate from the close contact with the soil, where manure or sewage sludge may represent a primary source of pathogenic microbes, together with irrigation water [7]. Besides pathogenic microbes, F&V usually harbor a complex microbial community that is influenced by several factors, such as the type of product and the cultivar, geographical area, agricultural practices, season and climate [8,9,10]. After harvesting, the microbiota may be affected by processing, handling and storage conditions [11,12,13]. In Western countries, F&V are often sold by large-scale retail after some pre-processing steps, including peeling, slicing, cutting, washing and packaging. This manipulation may cause the disruption of vegetable cell walls, leading to the release of juices rich in nutrients, that may quickly promote the microbial growth [14,15]. Besides this, the high water activity and neutral (many vegetables) or low acidic (many fruits) pH may represent the perfect environment for the development of several microbial populations, including human pathogens and spoilage microorganisms [16]. Indeed, it is estimated that 20–30% of F&V are lost due to spoilage, with microbial alteration contributing the most [17]. As resident microbiota is often responsible for post-harvest alterations [18], understanding its dynamics and how it is affected by processing and storage conditions is an important step towards the improvement of quality and safety management of fresh, minimally processed F&V [19].

Cultural-dependent studies discovered that bacteria more often involved in F&V spoilage belong to the genera *Corynebacterium*, *Pseudomonas*, *Erwinia* and other *Enterobacteriaceae*, while *Alternaria*, *Cladosporium* and *Botrytis* are frequently reported as eukaryotic spoiling microbes [20,21]. Interestingly, several spoilage mechanisms are common for both bacteria and fungi, such as the production of pectin methylesterases, a heterogeneous group of enzymes responsible for the hydrolysis of pectin, that is one of the main polysaccharidic components of plant cell walls [22,23]. Besides lysis of pectins, additional spoilage mechanisms have been described. In particular, enzymes responsible for cellulose disruption cause the release of glucose, and might enhance infection by plant pathogens. Once the F&V surface is damaged by microbial enzymes or by insect/human wounding, other spoilage taxa might penetrate into internal tissues, contributing to spoilage [22]. 

Microorganisms resident on the phyllosphere of F&V may resist the minimal processing applied post-harvesting and develop during the refrigerated storage, influencing product shelf-life and safety [24]. Despite the serious consequences of the multiplication of pathogenic and spoilage microorganisms on fresh F&V, a surprisingly limited amount of data has been published about their prevalence in products available to the consumers.

In this study, culture-dependent microbiological analysis and high-throughput sequencing (HTS) were used to explore the microbiota and mycobiota associated with different fresh, minimally-processed F&V products available in the market, i.e., at the end of all handling, packaging and transport phases. In particular, we collected F&V that are commonly consumed in Southern Italy, such as spinaches, lettuces, iceberg lettuces, valerian and arugula (which composed the “Green leafy vegetables group”), fennels, tomatoes and pears. Metagenomics may be useful to obtain a holistic view of the postharvest microbial populations in F&V, providing opportunities to identify previously unknown relationships between community members, including potential pathogens. Accordingly, characterization of the bacterial and fungal communities on fresh produce has the potential to support the development of targeted strategies to control the microbial composition of fresh F&V and limit the growth of spoilage/pathogenic taxa.

## 2. Materials and Methods

### 2.1. Samples Collection

A total of 129 fruit and vegetables samples, pre-packaged in packs of 200 g (subjected to a pre-treatment of sorting and coarse cleaning) and of Italian origin, were bought in several supermarkets located in Campania region (Southern Italy) from October 2019 to March 2020. All products belonged to different brands.

In particular, 47 samples were included in the “green leafy vegetables” group, which was composed of: lettuce (*Lactuca sativa*, *n* = 14), iceberg lettuce (*Lactuca sativa* var. *capitata*, *n* = 10), spinach (*Spinacia oleracea*, *n* = 3), arugula (*Eruca versicaria* subsp. *sativa*, *n* = 13) and valerian (*Valeriana officinalis*, *n* = 7).

The remaining samples were: 54 fennels (*Foeniculum vulgare*); 17 cherry tomatoes (*Solanum lycoperscium*) belonging to Ciliegino, Datterino, Campanino, Pachino and Yellow varieties; and 11 pears (*Pyrus communis*) belonging to Abate, Spadona and William varieties. Pears and tomatoes were included in the “Fruit” group, while green leafy vegetables and fennels were grouped as “leaves”, considering the edible part. All the samples were obtained from intact packaging.

After purchase, the products were stored at 4 °C simulating the home storage conditions. The analysis was made on the expiration date declared on the label.

### 2.2. Culture-Dependent Microbiological Analysis

A portion of F&V samples (about 20 g) were serially diluted in quarter strength Ringer’s solution (Oxoid, Basingstoke, UK), and after a homogenization step in a Stomacher (1 min at 230 rpm), dilutions were plated on different media: PCA (Plate Count Agar, Oxoid), VRBGA (Violet Red Bile Glucose Agar, Oxoid), TBX (Tryptone Bile X-GLUC, HiMedia, Mumbai, India), DRBC agar (Dichloran Rose Bengal Chloramphenicol, Oxoid) supplemented with 100 mg/L of chloramphenicol.

Plates were incubated at 20 °C (PCA both in aerobic and anaerobic conditions), 37 °C (VRBGA), 42 °C (TBX) and 28 °C (DRBC) for the count of total psychrotrophic (aerobic or anaerobic) populations, *Enterobacteriaceae*, *E. coli*, molds and yeasts, respectively. Colonies were counted after 48 h, except for yeasts and molds that were incubated for 5 days.

### 2.3. DNA Extraction and Amplicon Illumina Sequencing

Samples were weighted (Table S1) and transferred to a sterile bag, STE (100 mM NaCl, 10 mM Tris-HCl [pH 8.0], 1 mM EDTA [pH 8.0]) buffer was added in 5:1 ratio and microorganisms were detached from the surface of the F&V by shaking, without damaging the tissues and limiting the extraction of vegetable DNA. The STE solution containing the microorganisms was then collected and centrifuged at 13,000× *g* for 2 min and the cell pellets were collected and stored at −20 °C, until DNA extraction. Total DNA extraction from samples was carried out by using the DNeasy PowerSoil Pro Kit (Qiagen, Hilden, Germany), according to the manufacturer’s instructions and quantified using the NanoDrop spectrophotometer (NanoDrop Technologies, Inc., Wilmington, DE, USA).

The V3-V4 region of the 16S rRNA gene (∼460 bp) was amplified using primer S-D-Bact-0341F/S-D-Bact-0785R [25] and conditions previously reported [26]. The fungal diversity was studied by amplification of ITS1-2 region (200–450 bp) using primer EMP.ITS1f/EMP.ITS2r [27]. Amplicons were independently barcoded and then pooled in an equimolar pool using the Microlab STARlet workstation (Hamilton) according to the Illumina metagenomic sequencing library preparation protocol. Equimolar pools of amplicons were sequenced on a MiSeq platform, yielding 2 × 250-bp, paired-end reads.

### 2.4. Bioinformatics and Statistical Analysis

Bacterial and fungal reads were imported into QIIME 2 [28] (q2cli version 2020.11.1). Primers were trimmed and sequences were quality checked, denoised and merged through the plugin ‘dada2’, using parameters “--p-chimera-method pooled”, “--p-pooling-method pseudo”, “--p-min-fold-parent-over-abundance 10” and “--p-max-ee 2”. Bacterial and fungal representative sequences were mapped against the Greengenes 13_8 [29] and the UNITE 8.3 [30] databases, respectively. For both datasets, taxonomic assignment was carried out with the ‘feature-classify’ plugin (‘classify-consensus-vsearch’ method). Amplicon Sequence Variant (ASV) tables were collapsed at the genus level afterwards. Chloroplast contamination was removed from the ASV tables, and the relative abundance of other taxa was recalculated. Statistical analyses and plotting were carried out in R environment (http://www.r-project.org, accessed on 1 June 2022). Shannon and Simpson alpha-diversity indices were calculated through the function ‘diversity’, whereas Bray–Curtis distance matrices were computed using ‘vegdist’ (‘vegan’ R package). Principal Coordinates Analysis (‘cmdscale’ function from ‘base’ package) was carried out on the distance matrices, and plotting was carried out using the ‘ggplot2’ R package. Differences between the groups were further tested with Multivariate ANOVA (‘adonis’ function from ‘vegan’ R package). A pairwise Wilcoxon–Mann–Withney test (‘pairwise.wilcox.test’ function in package ‘base’) was used to assess significant differences in the alpha diversity indices or in the abundance of taxa between the groups. If not specified, *p*-value < 0.05 was considered statistically significant. Boxplots representing the abundance of microorganisms or the distribution of alpha diversity indices in each group were drawn with functions ‘geom_boxplot’ and ‘geom_jitter’ from the ‘ggplot2’ package. The ‘InteractiVenn’ web tool was used to obtain the Venn diagrams [31]. The Mantel test (function ‘mantel’ from ‘vegan’) with 10,000 permutations was used to assess the relationship between Bray–Curtis bacterial and fungal distance matrices. Correlations were computed with ‘cor.test’ from the ‘psych’ R package, and the FDR method was used to correct p-values, whereas the plot was obtained by ‘corrplot’ (‘corrplot’ package).

## 3. Results

### 3.1. Culture-Dependent Analysis

The microbial loads of the different populations counted were significantly different among the F&V types at the expiration date, as obtained by the analysis of variance (ANOVA; Table 1): yeasts and molds (*p* = 0.00495), total psychrotrophic anaerobic counts (*p* = 1.82 × 10^−11^), total psychrotrophic aerobic counts (*p* = 1.04 × 10^−14^) and *Enterobacteriaceae* (*p* = 9.19 × 10^−5^). *E. coli* was <10 CFU/g in all the samples.

Green leafy vegetables and fennels showed higher levels of yeasts and molds than tomatoes, possibly for the closer contact with soil in these products. Total psychrotrophic aerobic and anaerobic populations were significantly higher in green leafy vegetables and fennels compared to tomatoes and pears. Furthermore, green leafy vegetables showed higher levels of *Enterobacteriaceae* compared to all other products (Table 1).

### 3.2. Metagenomic Analysis of Bacterial and Fungal Populations

From the collapsed ASV table, a total of 726 genera were found, belonging to 20 phyla, i.e., Acidobacteria, Actinobacteria, Armatimonadetes, Bacteroidetes, Chlamydiae, Chlorobi, Chloroflexi, Cyanobacteria, Elusimicrobia, Fibrobacteres, Firmicutes, Gemmatimonadetes, Nitrospirae, Planctomycetes, Proteobacteria, Synergistetes, Tenericutes, Thermotogae, Verrucomicrobia and Thermi. In particular, Bacteroidetes and Proteobacteria were the most represented phyla (with 63 and 243 genera, respectively), as well as the most abundant, with the former ranging between 0.00–40.20% (mean relative abundance 12.29 ± 11.46%) and the latter between 21.97–100% (73.89 ± 17.82%).

**Figure 1:**
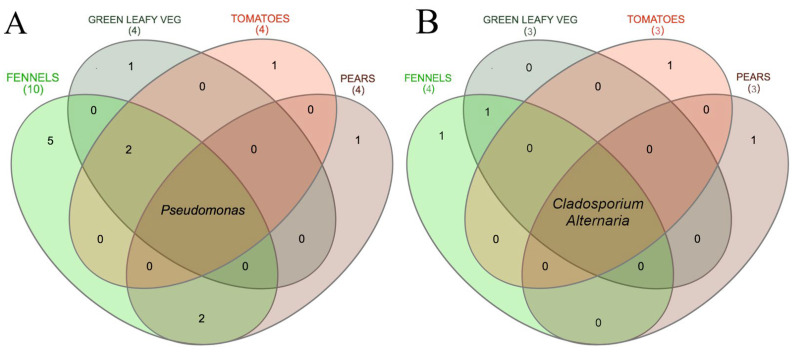
Venn diagrams showing the (**A**) bacterial and (**B**) fungal genera shared among different F&V. Only taxa with a relative abundance > 0.5% in at least 80% of samples in each type of F&V are included. The number of taxa retained after the filtering for each type is reported in parenthesis.

At genus level, the most abundant taxon was *Pseudomonas* (mean relative abundance 13.46 ± 14.94%), followed by an unassigned genus belonging to the family *Enterobacteriaceae* (9.48 ± 12.19%) and *Erwinia* (7.65 ± 13.81%). In addition, *Pseudomonas* represented the only bacterial genus with >0.5% abundance that was shared by all the F&V types (Figure 1A).

**Figure 2:**
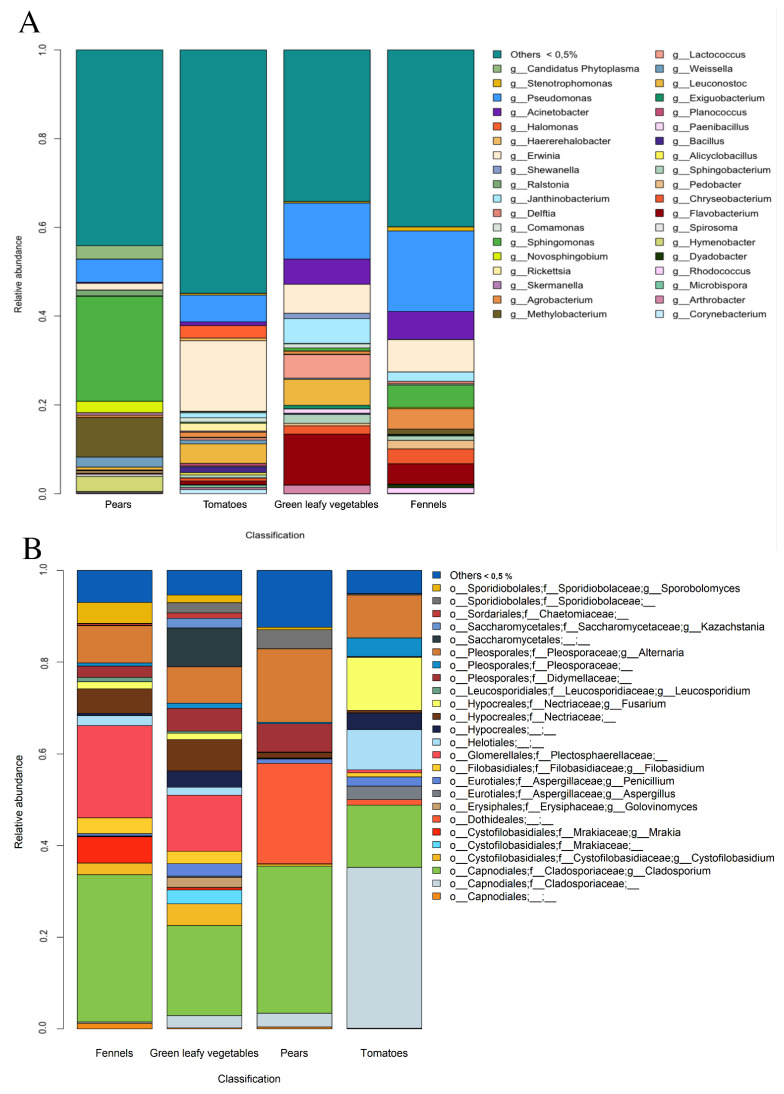
Barplots showing the mean relative abundance of (**A**) bacterial and (**B**) fungal genera within each type of F&V. Only taxa with a mean relative abundance > 0.5% are plotted.

**Figure 3:**
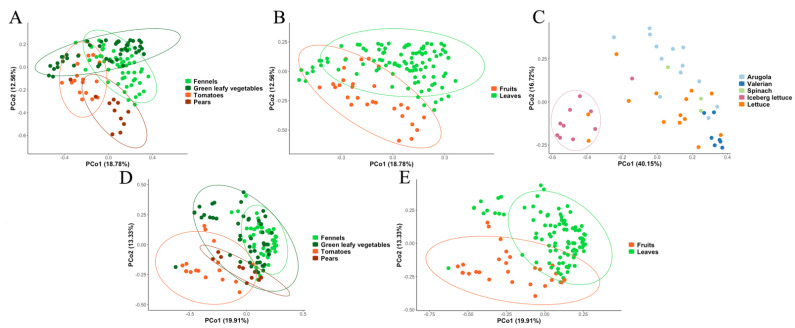
PCoAs based on Bray–Curtis distances matrices obtained from bacterial (**A**–**C**) and fungal (**D**,**E**) taxonomic profiles. Samples are colored according to the types of F&V (**A**,**D**) and the type of edible part (fruits or leaves; (**B**,**E**)). (**C**) PcoA including only green-leafy vegetable samples, colored according to the vegetable species.

Moreover, evaluating the average taxonomic composition of the microbiota, a high diversity was observed (Figure 2A). *Sphingomonas* (phylum *Proteobacteria*), *Methylobacterium* (phylum *Proteobacteria*) and *Pseudomonas* (*Proteobacteria*) were the most abundant taxa in pears, while *Pseudomonas*, *Erwinia* and *Halomonas* (*Proteobacteria*) were more abundant in tomatoes. Leafy vegetables and fennels showed a more similar microbiota compared to pears and tomatoes and *Pseudomonas*, *Acinetobacter*, *Erwinia* and *Flavobacterium* were the dominant taxa in both types. In addition, *Janthinobacterium*, *Delftia* and *Leuconostoc* were abundant in leafy vegs (3.06, 0.27 and 2.78%, respectively), while *Agrobacterium* and *Sphingomonas* were more abundant in fennels (2.38 and 4.42, respectively). These results were supported by PCoA analysis based on Bray–Curtis distance matrix, that showed a good separation between the four types of products (PERMANOVA *p*-value < 0.0001; Figure 3A), as well as between fruity and leafy vegetables (PERMANOVA *p*-value < 0.0001; Figure 3B). Moreover, when considering the different types of green leafy vegetables, iceberg lettuce samples clustered separately and clearly differed from the other samples in the same category (Figure 3C).

**Figure 4:**
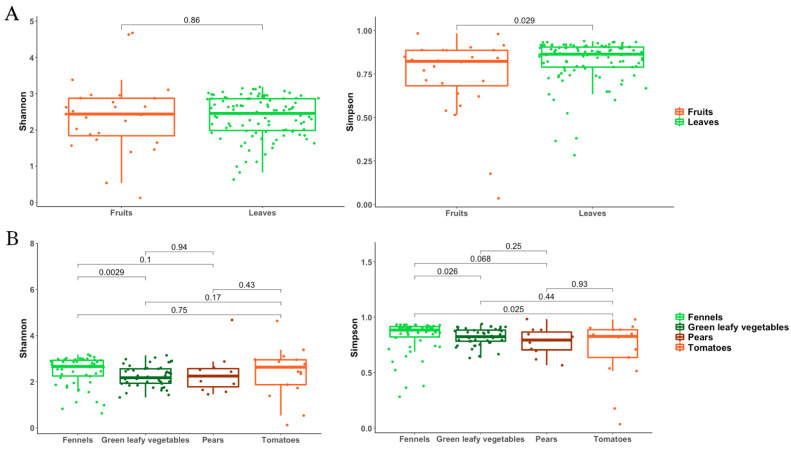
Bacterial alpha diversity indices compared between (**A**) fruits and leaves and (**B**) the four types of F&V.

Accordingly, the alpha diversity analysis (in particular Simpson’s diversity index) revealed a significantly lower biodiversity on the surface of fruits than on leaves (Figure 4A), while fennels showed a higher diversity compared to leafy vegetables and tomatoes (Figure 4B).

**Figure 5:**
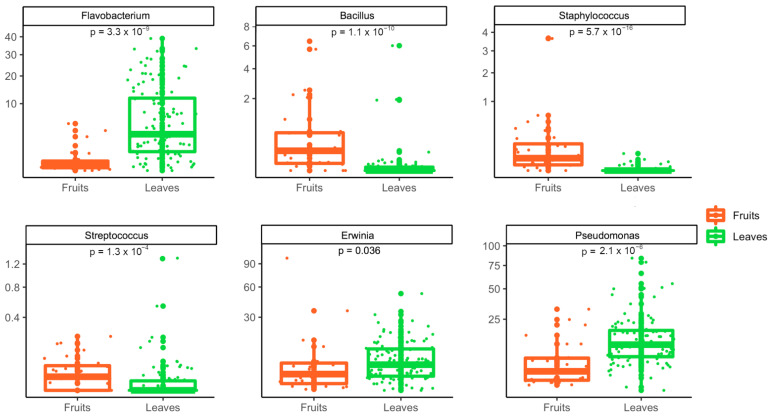
Bacterial genera showing a significant difference between fruits and leaves.

Finally, the relative abundance of the taxa was compared between leaves and fruits, in order to highlight statistical differences. Overall, 170 genera were differentially abundant between fruits and leaves (Table S2A). Among these, *Bacillus*, *Staphylococcus* and *Streptococcus* were more abundant on fruit surfaces, while *Flavobacterium*, *Erwinia* and *Pseudomonas* showed a significantly higher abundance on leaves (Figure 5).

A total of 353 fungal genera were identified, belonging to 8 phyla, i.e., Ascomycota, Basidiomycota, Chytridiomycota, Mortierellomycota, Mucoromycota, Olpidiomycota, Rozellomycota and Zoopagomycota. Ascomycota and Basidiomycota were the most represented phyla (with 207 and 133 genera, respectively), as well as the most abundant, with the former ranging between 0.06–99.77%, and the latter between 0.00–62.81%. 

At genus level, the most abundant taxon was *Cladosporium* (mean relative abundance 25.15 ± 16.9%), followed by an unassigned genus belonging to the family of *Plectosphaerellaceae* (12.97 ± 15.87%) and *Alternaria* (8.89 ± 9.21%). In addition, *Cladosporium* and *Alternaria* represented the core mycobiota, since they were the only genera that had a relative abundance higher than 0.5% in at least 80% of all the samples (Figure 1B).

Moreover, differences in the average taxonomic composition were observed between the types of products (Figure 2B). Fennels and leafy vegs showed a more similar mycobiota, with high abundance of *Plectosphaerellaceae* (20.1 and 12.3%, respectively), *Nectriaceae* (5.3 and 6.8%) and *Filobasidium* (3.5 and 2.7%). In addition, *Mrakia* and *Sporobolomyces* were more abundant in fennels, while *Penicillium*, *Didymellaceae*, *Kazachstania* and another genus of *Saccharomycetales* showed higher abundance in leafy vegs. On the contrary, pears were characterized by high abundance of unidentified *Dothideales* (21.9%), while *Cladosporiaceae* (35.1%), *Helothiales* (8.8%) and *Fusarium* (11.6%) were found at high levels in tomatoes (Figure 2B). PCoA based on Bray–Curtis distance matrix showed a good separation between the four types of F&V (PERMANOVA *p*-value < 0.0001; Figure 3D), as well as between fruits (pears and tomatoes) and leaves (leafy vegetables and fennels; PERMANOVA *p*-value < 0.0001; Figure 3E).

**Figure 6:**
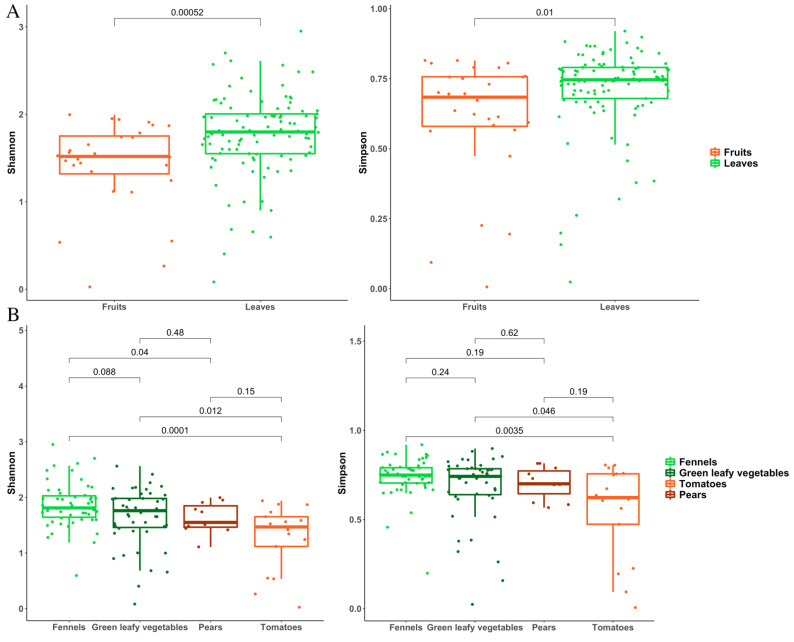
Fungal alpha diversity indices compared between (**A**) fruits and leaves and (**B**) the four types of F&V.

As already shown for the bacterial populations, also the mycobiota of leaves showed a higher diversity compared with fruits (Figure 6A), whereas no difference was highlighted comparing the four types of vegs (Figure 6B).

**Figure 7:**
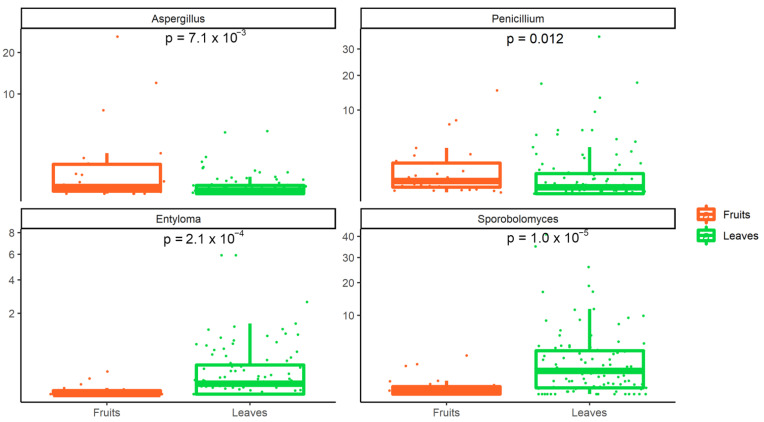
Fungal genera showing a significant difference between fruits and leaves.

Finally, the relative abundance of the taxa was compared between leaves and fruits, to highlight differences in the mycobiota composition. Overall, 67 fungal genera were differentially abundant between fruits and leaves (Table S2B). Among these, *Aspergillus* and *Penicillium* were more abundant on fruits surfaces (with *Aspergillum* more abundant on tomatoes than pears), whereas *Entyloma* and *Sporobolomyces* showed a significantly higher abundance on leaves (both being more abundant on fennels surface than on leafy vegetables; Figure 7).

### 3.3. Bacterial and Fungal Communities Are Correlated

**Figure 8:**
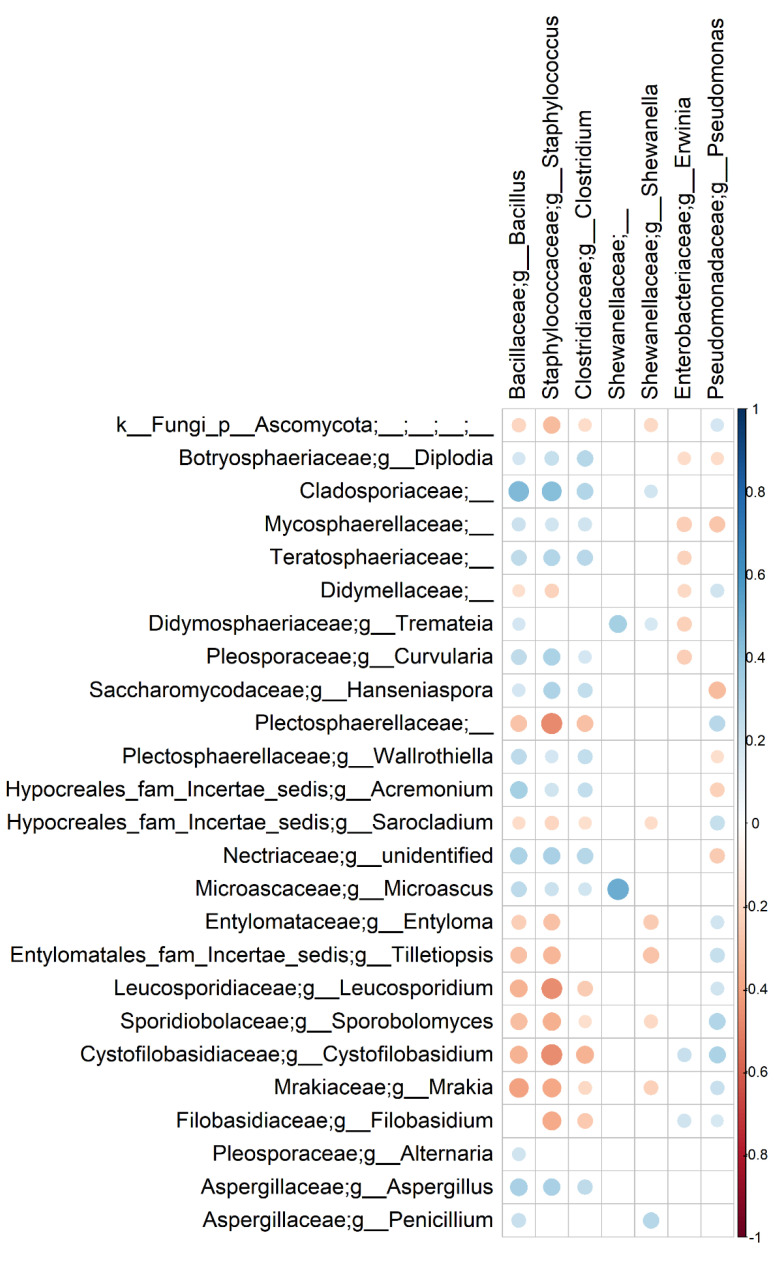
Spearman’s rank correlation matrix of bacterial and fungal taxa. Strong correlations are indicated by large circles, whereas weak correlations are indicated by small circles. The colors of the scale bar denote the nature of the correlation, with 1 indicating a perfectly positive correlation (dark blue) and −1 indicating a perfectly negative correlation (dark red) between two microbial taxa. Only significant correlations (FDR < 0.05) are shown.

The Mantel test with 10,000 permutations was used on the Bray–Curtis distance matrices to test whether the bacterial and fungal communities composition were correlated. The matrices had a Mantel statistic r = 0.40 (*p*-value < 0.001), suggesting that the communities are slightly and positively correlated. Hence, we further explored correlations between bacterial and fungal genera across all the groups of samples. Although Spearman’s correlations between bacteria and fungi mainly involved some minor taxa, we observed that *Aspergillus* abundance was positively correlated with *Bacillus*, *Staphylococcus* and *Clostridium*, whereas *Pseudomonas* co-occurred with both *Entyloma* and *Sporobolomyces* (Figure 8). In addition, *Bacillus* and *Staphylococcus* co-excluded with several fungal taxa (e.g., *Mrakia*, *Sporobolomyces*, *Plectrosphaerellaceae* and *Leucoporidium*), highlighting possible antagonistic dynamics among these taxa. Finally, *Penicillium* co-occurred with both *Bacillus* and *Shewanella*. No correlation was observed for *Cladosporium*. All the correlations had an FDR corrected *p*-value < 0.05. 

## 4. Discussion

Fruit and vegetables constitute an important food group, and its consumption has been positively linked with health [32,33]. They can be considered as “functional foods” because their balanced mixtures of phytochemicals make them protective against various diseases such as arthritis, cancer, diabetes, as well as aging [34]. 

Different F&V present diverse surface morphology, tissue composition and metabolic activities that make each product as a unique ecological niche, selective for specific microbial groups [35]. These factors might explain the significantly different biodiversity that we observed between fruits and vegetables (Figure 4 and Figure 5).

F&V may be contaminated by spoilage and pathogenic microorganisms (bacteria or fungi) at any stage from production to consumption [35,36]. The contamination most frequently occurs in the field or during post-harvest handling [37] and potential sources include animals, feces, soil, dust, irrigation water, insecticides, fungicides, inadequately composted manure and human handling [35,37]. Besides pathogenic microorganisms (e.g., *E. coli*, *Salmonella*, *Listeria monocytogenes* and *Campylobacter jejuni* [37]), spoiling microbes can rapidly grow on the damaged surfaces of F&V, which release juices rich in nutrients [35]. In addition, some fungal taxa may metabolize organic acids present in these exudates, increasing the pH and promoting the growth of bacterial pathogens [35]. Leff and Fierer [9] demonstrated that fruits and vegetables contain a wide diversity of bacterial and fungal taxa that vary significantly according to the product types. Exploring the microbial diversity on the surface of F&V represents the first step to control their proliferation, increasing the product shelf-life and improving quality and safety [35,37]. 

Moreover, the inefficiency of home washing in microorganisms removal was highlighted [38], demonstrating the responsibility of producers and distributors to guarantee products hygienic quality [39].

Our results demonstrated that F&V harbor complex bacterial and fungal communities, dominated by the phyla *Bacteroidetes*/*Proteobacteria* and *Ascomycota*/*Basidiomycota*, respectively. This result is largely consistent with those found in previous studies. In particular, *Ascomycota* and *Basidiomycota* have been reported as dominant phyla on the phyllosphere/surface of several plants and fruits, such as grapes, tomatoes, cherries and spontaneous grasses [40,41,42,43]. Moreover, *Bacteroidetes* and *Proteobacteria* were reported as the most abundantly represented bacterial phyla on lettuce [44]. This is also consistent with results reported for other plant-associated microbial communities, such as grape [45], various tree species [46] and fresh spinaches [47].

Members of the *Enterobacteriaceae* family dominated the microbiota of vegetables, while they were found at lower abundance on fruits: this suggests that specific vegetable host–bacterium interactions potentially driven by intrinsic factors (such as F&V surface shape and composition, presence of antimicrobial compounds) may result in variations in the abundance of some taxa, that become discriminant of the different vegetables [48]. Relative abundances of potentially pathogenic and spoilage bacterial and fungal genera were also assessed. Both *Staphylococcus* and *Pseudomonas* showed significant differences between leafy vegs and fruits. In particular, *Staphylococcus* was more abundant on fruit surfaces and its presence could be an indicator of human contamination (cross-contamination): indeed, pears and tomatoes are more exposed to human contact during the harvest, while washing operations for leafy vegetables and human contact with non-edible part (stem) for fennels could explain the reduced abundance of *Staphylococcus* in these products. 

Staphylococci are commonly distributed in the environment, animals and humans, and *Staphylococcus aureus* is known as a food-borne pathogen producing heat-stable enterotoxins that cause food poisoning. Some reports indicated that *S. aureus* was frequently isolated from lettuce, fruits and sprouts [49]. On the other hand, members of the genus *Pseudomonas* showed a significantly higher abundance on leaves. Most *Pseudomonas* species are psychrotolerant or psychrotrophic (growing below 15 °C), explaining their dominance on refrigerated products [50]. Due to their simple nutritional requirements and their high metabolic versatility, these bacteria are ubiquitous, and have been isolated from a variety of sources (soil, fresh water, humans, plant and animal surfaces, cosmetics, medical products and instruments, foods). According to our results and previous reports [51,52], the vegetables cultivated in close contact with soil may be more easily contaminated with *Pseudomonas* spp. Some *Pseudomonas* species may act as opportunistic pathogens in animals and humans, or as phytopathogens. In addition, some species are involved in off-flavor release, due to the production of volatile compounds and amino acid metabolites [50,53,54].

The analysis of the fungal genera revealed that *Aspergillus* and *Penicillium* are significatively more abundant on the surface of fruits (pears and tomatoes; Figure 7).

Some species may produce toxic secondary metabolites, mainly in the post-harvest phase, which represent a health risk [55,56,57]. In particular, *Aspergillus* spp. might produce cancerogenic mycotoxins, i.e., aflatoxins, ochratoxin A (OTA) and sterigmatocystin, during the developmental stages of fruits [58]. In addition, several *Aspergillus* species are associated with fruit rottenness, contributing to their loss. Likewise, *Penicillium* spp. have been reported as major spoilage microorganisms for tomatoes and pears [59,60].

On the other hand, *Entyloma* and *Sporobolomyces* were among the taxa that discriminated the leaves from the fruit mycobiota. In particular, members of the genus *Entyloma* are of particular concern, since they are reported as one of the most relevant causes of smut, with the formation of white spots on the leaves of *Apiaceae*, which include fennels [61]. *Sporobolomyces*, which showed a mean relative abundance of 3.19% in the leafy vegetables, has been reported as part of the core mycobiota of broccoli phyllosphere [11] and rice paddy [62], but it was also linked with several human diseases, such as dermatitis, allergic alveolitis and cerebral infections [63]. Although being considered as a rare invasive yeast [64], the wide distribution of members of this genus and the high abundance that it can reach on fresh leaves that are usually consumed raw, make it potentially dangerous.

Microbiota composition among the different green leafy vegetables revealed that iceberg lettuce samples clearly differed from the rest of leafy products. This suggested that the particular leaf morphology may influence the bacterial population: iceberg lettuce heads are more closed and compact compared with other lettuce types, and this could protect them from microbial soil contamination. Furthermore, if stored in suitable conditions, they present longer shelf-life than other lettuce types.

In addition, the analysis of the core mycobiota and microbiota shared by all samples highlighted that some alterative and potentially pathogenic *taxa* dominate the communities of all the products. Indeed, *Cladosporium* and *Alternaria* were the core fungal genera (Figure 1B). These *taxa* have been reported frequently as a cause of rottenness and plant disease, and [65] also highlighted the role of the market environments’ airborne spores in contaminating fruits and vegetables. Besides their spoilage role, microorganisms belonging to these genera might also represent a health threat. For example, *Cladosporium* spp. and *Alternaria* spp. spores are among the causes of allergic diseases or sensitizations [66], and an increasing trend of the incidence of these symptoms was recently claimed [67]. Similarly, *Pseudomonas* represented the core bacterial taxon. It has been demonstrated that the predominance and persistence of pseudomonads in foods and on surfaces of food processing plants is related to the ability of these microorganisms to form biofilm, which enhances their resistance to adverse conditions, including several antimicrobial treatments [50]. 

Furthermore, our results suggest that bacterial and fungal communities may interact in F&V, leading to synergistic or antagonistic dynamics. Bacteria and fungi are known to interact and co-evolve in several environments [68], although a very few studies focused on interactions on fresh F&V. Interestingly, *Pseudomonas* showed a positive correlation with several fungal taxa that were reported to be associated with spoilage, such as *Sporobolomyces* and *Entyloma*, whereas *Aspergillus* co-occurred with *Staphylococcus*, *Clostridium* and *Bacillus*. Mechanisms underlying such patterns are not clear, and these correlations might merely depend on the fact that these taxa share the same natural habitat. However, further efforts in deciphering interactions between bacteria and fungi on fresh F&V might be useful to adopt specific and focused biocontrol procedures. 

Taken collectively, our results suggest that the microbiota and mycobiota of fruit and vegetable products are characterized by high biodiversity and that their composition is extremely variable depending on the product type. The type of the edible fraction (fruit or leaf) and the particular leaf morphology (for green leafy vegetables) represent discriminating factors in selecting specific bacterial and fungal populations, and they might also have an effect on the whole biodiversity of the communities. Indeed, leafy vegetables (fennels, green leaves) grow closer to the soil compared to fruits. However, since we lack information about the microbial composition of the soil where F&V were cultivated and about the specific handling, washing, transport and storage process, we are not able to assess the influence of these factors on the microbiota inhabiting the final product, which might be relevant [11,12,13]. Additionally, we observed that several members of both communities resist on the surface of the food product for the whole shelf-life, potentially causing spoilage, and, in some circumstances, representing a safety hazard. Since the inefficiency of home washing in removing microorganisms from F&V surfaces has been highlighted [38], it is important to understand the complex microbial ecosystem that is unique for each product, in order to establish specific control measures that can be applied during harvesting, handling and distribution to the final consumer.

Hence, further efforts from researchers and food industry are needed to explore the biodiversity and the product-specific microbial community, in order to elaborate and validate such control measures. 

## Figures and Tables

**Table 1:** Microbial loads (log CFU/g) of yeasts and molds, total psychrotrophic anaerobic and aerobic populations, *Enterobacteriaceae*. The values shown are the mean value of all the samples analyzed for each product type (±standard deviation). Different letters in column indicate significantly different values between the different product types, as determined by paired *t*-tests (*p* < 0.05).

	Yeasts and Molds (28 °C)	Total Psychrotrophic Anaerobic Counts (20 °C)	Total Psychrotrophic Aerobic Counts (20 °C)	Enterobacteriaceae (37 °C)
Green leafy vegetables (*n* = 47)	4.20 ± 0.82 ^a^	7.27 ± 0.96 ^a^	7.56 ± 0.48 ^a^	4.53 ± 0.89 ^a^
Tomatoes (*n* = 17)	2.99 ± 0.72 ^b^	4.13 ± 0.96 ^b^	4.38 ± 1.14 ^b^	2.30 ± 1.16 ^b^
Fennels (*n* = 54)	3.91 ± 1.66 ^a^	6.47 ± 1.23 ^a^	6.74 ± 0.82 ^a^	3.49 ± 1.20 ^b^
Pears (*n* = 11)	3.38 ± 0.72 ^a,b^	4.20 ± 0.88 ^b^	4.29 ± 0.83 ^b^	1.32 ± 1.94 ^b^

## Data Availability

The raw sequence reads generated in this study have been deposited in the Sequence Read Archive (SRA) of the NCBI under accession number PRJNA835434.

## Supplementary Materials

The following supporting information can be downloaded at: https://www.mdpi.com/article/10.3390/foods11142164/s1, Table S1: Information about the product sampled parts and the weight used for DNA extraction of each product, Table S2A: Mean relative abundance (%) of differentially abundant bacterial ASV between vegetables and fruits, Table S2B: Mean relative abundance (%) of differentially abundant fungal ASV identified in vegetables and fruits.
